# Swabs versus native specimens in severe head and neck infections: a prospective pilot study and suggestions for clinical management

**DOI:** 10.1007/s10006-025-01382-y

**Published:** 2025-04-14

**Authors:** Norbert Neckel, Christin Ohm, Oliver Wagendorf, Ulrike Kielburg, Daniel Tröltzsch, Jonas Wüster, Saskia Preißner, Francesca Ronchi, Benedicta Beck-Broichsitter, Max Heiland, Susanne Nahles

**Affiliations:** 1 Department of Oral and Maxillofacial Surgery, Charité—Universitätsmedizin Berlin, Corporate Member of Freie Universität Berlin, and Humboldt-Universität Zu Berlin, Hindenburgdamm 30, 12203 Berlin, Germany; 2 Department of Oral- and Craniomaxillofacial Surgery, Faculty of Medicine, Medical Center, University of Freiburg, Freiburg, Germany; 3 Institute of Microbiology, Infectious Diseases and Immunology (I-MIDI), Charité-Universitätsmedizin Berlin, Humboldt-Universität Zu Berlin, and Berlin Institute of Health (BIH), Berlin, Germany; 4 Department of Oral and Maxillofacial Surgery, Katharinenhospital Klinikum Stuttgart, Kriegsbergstraße 60, 70174 Stuttgart, Germany

## Abstract

**Introduction:**

Head and neck infections, particularly odontogenic infections, can lead to serious complications if not properly managed. While swabs are commonly used for microbial identification, their reliability in polymicrobial infections is debated. This study evaluates the advantages of native tissue samples over swabs in the management of severe head and neck infections.

**Material and Methods:**

This prospective cohort study included patients with severe acute head and neck infections requiring hospitalization, surgical drainage, and microbiologic analysis. Swabs and native tissue/fluid samples were collected for pathogen cultivation, Gram staining, and resistance testing. Clinical data, infection characteristics, and antimicrobial resistance profiles were analyzed using descriptive and inferential statistics.

**Results:**

60 patients, 55% male (45.7 years) and 45% female (48.1 years) were analyzed. After antibiotic treatment, CRP and leukocyte levels decreased significantly, with higher CRP correlating with longer hospital stays. ICU admission correlated with hospital stay > 7 days. More Actinomyces and fungal species were identified in native tissue samples and more Streptococci in swabs. Antibiotic resistance, especially to clindamycin (1/3 of the cases), was associated with longer hospital and ICU stays. Clindamycin resistance correlated with increased ICU admission, while metronidazole resistance (10% of the cases) was associated with longer ICU stays. ICU admission was also associated with higher Cormack-Lehane scores.

**Conclusion:**

Severe head and neck infections require a comprehensive multidisciplinary approach. Native tissue should be obtained whenever possible. While microbiological findings varied between sampling methods, native samples may provide a broader spectrum of detected pathogens, which could be relevant for infection management. Given the increasing resistance to clindamycin, its indications should be critically re-evaluated. The implementation of targeted antimicrobial strategies and a risk-based classification system may help optimize patient management and improve outcomes.

## Introduction

Putrid infections of the head and neck area are very common [1]. They can originate from a variety of sources and tissues [1–4]. However, the most common source of deep head and neck infections is odontogenic [5, 6]. In general, early and aggressive local treatment and elimination of the focus is sufficient to eliminate the infection and prevent spread to the deep cervical regions [6–8]. However, if neglected or inadequately treated, these can lead to serious complications and even life-threatening clinical situations [7].

Early disease control is crucial to preventing complications. While minor infections in otherwise healthy individuals may not always require microbiological sampling for medical and economic reasons [8–10]. This differs for severe or prolonged treatment-resistant infections, because the clinical course is not always clear in advance. Therefore, early microbiologic sampling prior to antibiotic administration may be extremely helpful in later stages, although initial antimicrobial therapy must be empiric [11]. Early detection of adverse antimicrobial resistance can still prevent prolonged treatment failure in severe acute infections or long-term complications such as chronic osteomyelitis [12, 13]. Therefore, it is necessary to identify head and neck infections with a tendency to spread locally or systemically [14]. Furthermore, this tendency to spread seems to coincide with antibiotic resistance of the responsible microorganisms and has been described as an indication for hospitalization by Heim et al. [14]. Despite innovations like vortexing and sonication, traditional cultivation remains standard [15].

Given this, the options of obtaining native samples (NS), including the collection of pus or traditional swabs (S) are contrasted. While native samples are the most reliable source for microbiologic testing, they carry the risk of injuring critical structures such as the marginal mandibular branch of the facial nerve [16, 17]. On the other hand, aspirates from needle punctures sometimes do not yield enough pus or the specimen is still too viscous to aspirate [18]. Swabs, although easy to use, have been shown to yield less specimen material and microorganisms than aspirates and native specimens [14, 16, 17, 19]. This is particularly problematic in a polymicrobial, partially anaerobic microbial community with a variety of opportunistic bacteria and overlap with non-critical commensals [17]. Therefore, the Infectious Diseases Society of America and the American Society for Microbiology do not recommend swabs as the specimen of choice for soft tissue head and neck infections [17]. In fact, swabs may not yield any results at all, even in clinically advanced infections [14, 20, 21]. Indeed, Heim et al. found that 34.6% of the swabs showed no bacterial culture growth [14].

Despite consensus on the superiority of native specimens, swabs remain common in clinical practice and guidelines [14, 16, 19, 22, 23].

The aim of this prospective study was to evaluate the real-world applicability of native specimens versus swabs in severe head and neck infections. We also propose a treatment algorithm for the evaluation and management of these serious infections.

## Materials and methods

### Study protocol

This comparative prospective cohort study conforms to the Declaration of Helsinki and the European Medicines Agency guidelines for good clinical practice. The study protocol was approved by the Ethics Committee of the Charité-Universitätsmedizin Berlin, Germany (EA A2/193/19). The study was conducted at the Department of Oral and Maxillofacial Surgery of the Charité-Universitätsmedizin Berlin, Germany.

### Patients

**Fig. 1:**
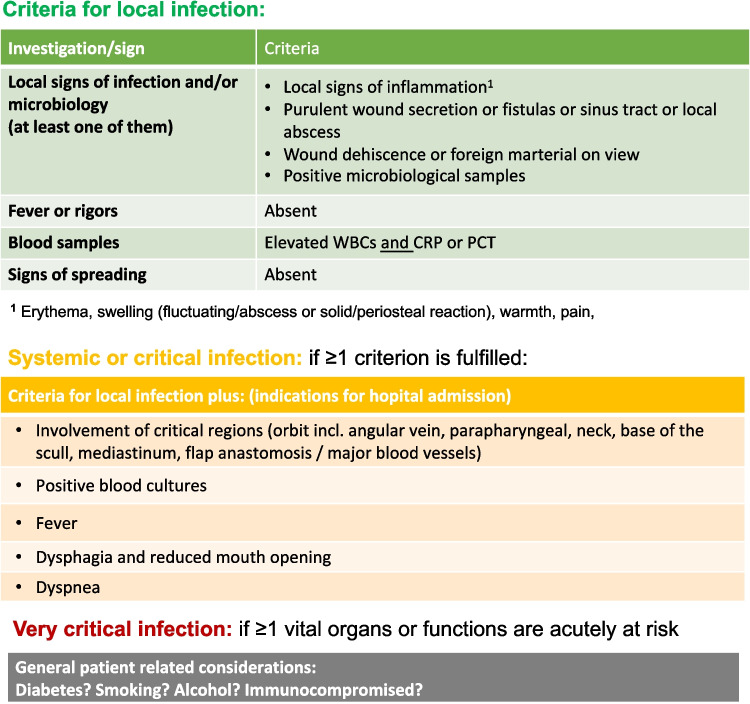
Criteria for cranio- and maxillofacial infections – hospitalization needs to be considered in systemic and critical infections and is obligatory in very critical infections

60 patients were enrolled, all of whom had an indication for hospitalization as defined in Fig. 1. All underwent surgical drainage with microbiological sampling and antimicrobial susceptibility testing, 56 of whom consented to inpatient admission. For each patient, swabs and native tissue and/or fluid samples were collected and analyzed. Patient-specific factors such as age, sex, medical history, and length of the hospital stay were documented and analyzed. Medical history included cardiovascular, renal, hepatic and pulmonary disease, diabetes, immunodeficiency (due to medication or disease), smoking, nutritional status, and alcohol and drug abuse. The distinction between risk factors and pre-existing conditions is shown in Table 1. In addition, the location and extent of the infection, the primary cause or focus of the abscess, and diagnostic parameters such as white blood cell (WBC) count, C-reactive protein (CRP), and radiologic imaging were included in the analysis. CRP levels of more than 50 mg/L were considered “high” as these are considered to suggests bacterial infection [24]. Furthermore, anesthesiologic factors such as Mallampati score, Cormack-Lehane-Score and mouth opening after relaxation and method of intubation were evaluated.

**Table 1:** Synopsis of general patient characteristics

Variable		Case (*n* = 60)
Gender, n (%)	Male	33 (55,0%)
	Female	27 (45,0%)
Mean ages ± SD, years	Male	45,7 ± 13,4
	Female	48,1 ± 15,7
Risk factors, n (%)	Smoking	28 (46,7%)
	Alcohol	8 (13,3%)
	Other drugs	6 (10%)
	Obesity	9 (15%)
Pre-existing conditions, n (%)	Heart disease	11 (18,3%)
	Bronchial asthma	10 (16,7%)
	Diabetes type II	4 (6,7%)
	Kidney disease	3 (5%)
	Liver disease	2 (3,3%)
	Immunodeficiency^a^	6 (10%)
Other factors	CRP value on admission ± SD, (mg/L) CRP value upon discharge ± SD, (mg/L)	126,06 ± 89,26 50,92 ± 40,47
	WBC count on admission ± SD, (WBC × 10^9^/L) WBC count upon discharge ± SD, (WBC × 10^9^/L) Length of hospital stay (LOS) ± SD, days^b^	14,76 ± 6,11 8,79 ± 4,05 6,2 ± 3,8
	ICU stay, n (%)	8 (13,3%)
	Tracheostomy, n (%)	5 (8%)

case = 6 of which 2 × leukemia/stem cell transplantation, 2 × patient after chemotherapy, 1 × patient under chemotherapy, 1 × HIV

4 patients were not hospitalized

### Inclusion and exclusion criteria

Inclusion criteria were age ≥ 18 years, indication for surgical drainage, need for hospitalization (hospitalization criteria as shown in Fig. 1), and the informed patient consent to participate in the study. Patients were excluded if they were under the age of majority, under legal guardianship, or refused to participate in the study.

### Surgical treatment and microbiological sampling

Microbiological sampling was performed after disinfection with povidone-iodine solution (Braunol®, B. Braun SE, Melsungen, Germany), if surgery was performed under general anesthesia. In the few cases (n = 4) that were treated under local anesthesia, a mouthwash with 0.2% chlorhexidine solution (Chlorhexamed FORTE, GlaxoSmithKline Consumer Healthcare, London, United Kingdom) was used for one minute as an alternative to achieve intraoral germ reduction. After incision and surgical opening of each abscess, fluid aspiration was performed using a syringe with tips of different sizes depending on the viscosity. Air was immediately removed and the syringe was tapped as described by Murray [19]. The abscess cavity was then swabbed and the tip was placed in aerobic/anaerobic Amies transport medium (eSwab™, Copan Diagnostics Inc., USA). If easily accessible or if no fluid could be collected, native tissue samples were taken from the affected area without extending the surgical approach and stored and transported in a sterile container as recommended [16].

Intravenous (i.v.) antibiotics were administered only after sample collection. According to the German guidelines for odontogenic infections, the standard antibiotics administered were ampicillin/sulbactam (2 g/1 g, 3 × per day and, as a first alternative in case of allergy or intolerance, clindamycin (600 mg, 3 × per day) [22].

### Processing of the samples

Schematic Representation of the Microbiology Laboratory Sample Collection and Processing Workflow (Flow Diagram).

1. Collection of at least one swab and one native specimen each—the location of the abscess was categorized intraoperatively by the surgeon.

2. Transfer to an appropriate specimen container and transport to the microbiology laboratory (same day specimen submission and isolation of individual pathogen strains from specimens).

3. Cultivation of pathogens on culture media, parallel evaluation of Gram stain. (Culture media: Blood agar (CO2), UTI (urinary tract infection) agar, Candida/Sabouraud agar and CNA (colistin-nalidixic acid) agar). For anaerobic culture, Schaedler agar (with and without CV) and cooked blood agar (CO_2_) were used.)

4. Incubate in an incubator at 37 °C for 48 h.

5. Isolation of individual bacterial strains (via 3-streak isolation), identification of pathogens using MALDI-TOF and Vitek 2 methods.

Bacterial cultures remain in the incubator for 48 hours, after which the results are reported. Due to the special considerations in head and neck infections (e.g. bones, deep wounds/abscesses), long-term anaerobic incubation for 14 days was performed.

6. Antimicrobial susceptibility testing (the standard antimicrobial susceptibility testing method was Vitek2, with E testing and MIC determination used in certain cases, especially for multi-resistant pathogens. Testing is typically performed on the better cultured sample and the entire process takes approximately 48–72 h from sample receipt).

### Statistical analysis

The data collected included age, sex, pre-existing conditions, risk factors, abscess location and cause (e.g. focus tooth), length of hospital stay, identified pathogens, and resistance test results. Given the heterogeneity of the data, a descriptive statistical analysis was performed using Excel (Office 365, Microsoft, Redmond, USA) and SPSS 27 (IBM, Armonk, USA).

Frequency analyses were conducted for categorical variables, while for metric variables statistical measures such as the mean, median, standard deviation, quartiles, and range were calculated. Relationships between categorical variables were analyzed by cross-tabulation using Pearson’s chi-square test or Fisher’s exact test, depending on the data.

Metric variables were tested for normal distribution. Parametric tests, such as the t-test for two-sample comparisons and one-way ANOVA for multiple groups, were used when the data followed a normal distribution. For non-normally distributed data, the Wilcoxon signed rank test for dependent samples was used.

Significance was determined at a level of p < 0.05, meaning that results with a probability of error of less than 5% were considered statistically significant.

## Results

60 patients (33 males and 27 females) were included in this single-center prospective study. The mean age was 45.7 years in the male group and 48.1 years in the female group. 56 patients were treated under general anesthesia and 4 patients under local anesthesia. In 42 patients, abscess incisions were performed solely extraorally (transcervical or transfacial); in 11 cases, a combined intraoral and extraoral approach was used; and in 7 cases, only intraoral incisions were necessary. In 13 patients it was possible to sample pus as well as native tissue, while in 29 patients only pus and in 18 patients only native tissue samples were collected.

**Fig. 2:**
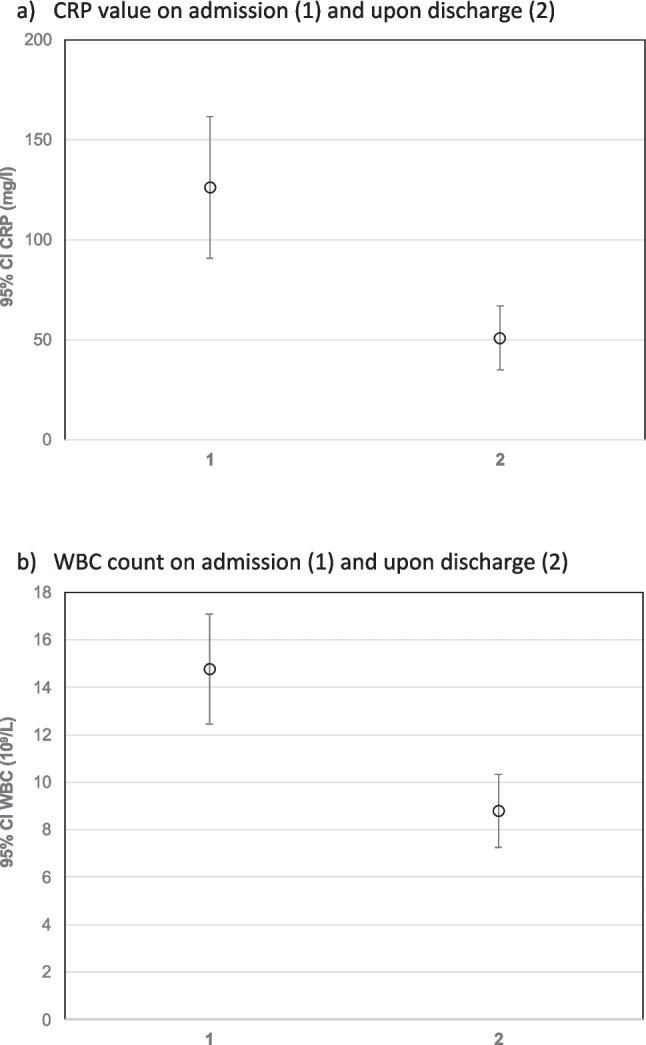
Comparison of CRP-values and leukocytes counts upon admission and discharge (**a**) CRP value on admission (1) and upon discharge (2) (**b**) WBC count on admission (1) and upon discharge (2)

**Fig. 3:**
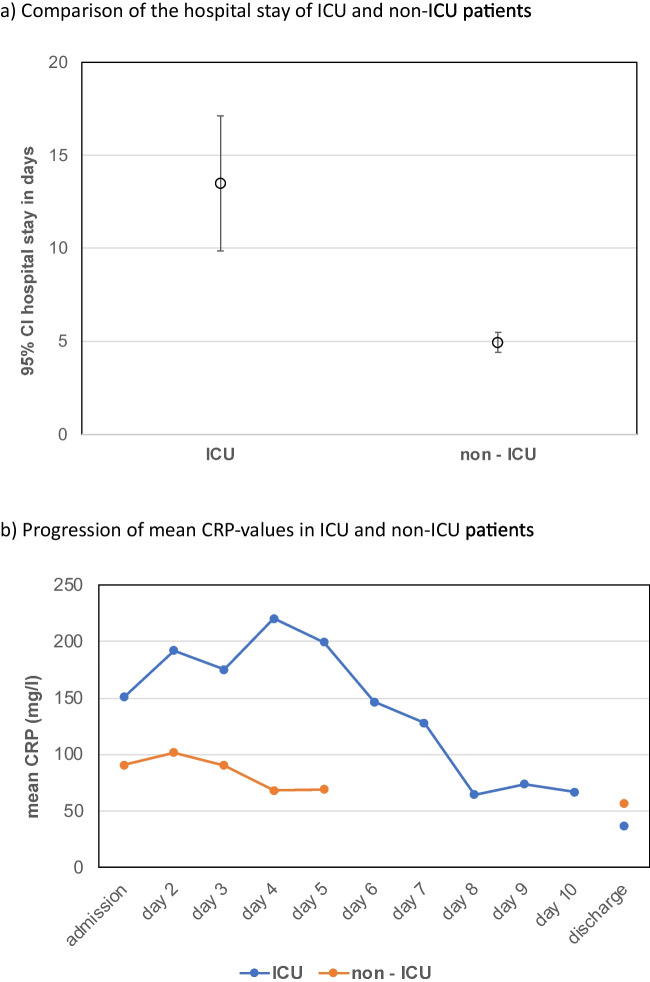
(**a**) Comparison of the hospital stay of ICU and non-ICU patients (**b**) Progression of mean CRP-values in ICU and non-ICU patients

20 patients had one risk factor and 12 patients had more than one risk factor, while 28 patients had no potentially relevant risk factors. 31 patients had no pre-existing medical conditions, 21 patients had one, and 8 had more than one. 30 patients had an indication for a computed tomography (CT) scan, either because of uncertainty about the origin or extent of the infection. 18 patients were referred to the hospital after the initiation of oral antibiotics in external outpatient clinics or practices. 10 of the 60 patients required a second look under general anesthesia. 6 of these patients were also admitted to the intensive care unit (ICU). One patient had a recurrent abscess after 6 months. The distribution of risk factors and comorbidities is shown in Table 1. The symptoms and abscess localization at initial presentation are summarized in Table 2. 70% of deep space infections correlated with CRP levels greater than 50 mg/l at initial presentation. Patients requiring a longer hospital stay had higher admission CRP values (p = 0.060). CRP levels on admission decreased significantly after surgical and antibiotic intervention (p = 0.001). Leucocyte levels also decreased significantly with treatment (p < 0.001). The distribution of inflammatory parameters on admission and at discharge (CRP and leukocyte count) is shown in Figs. 2 and 3. Furthermore, CRP levels were significantly higher in patients requiring ICU admission than in other hospitalized patients (Fig. 3). Abscess localization did not correlate with length of hospital stay, number of medications taken, microbiota or antibiotic resistance found in S and NTS. However, the longest hospital stay was recorded in a patient with a pterygomandibular abscess (19 days), followed by a case of a floor-of-mouth abscess (18 days), a patient with a submandibular abscess (15 days), and a patient with a perimandibular abscess (14 days). The aforementioned abscess localizations with the longest hospital stay were also the most common ones in this study.

**Table 2:** Symptoms and localization

	Frequency	Restricted mouth opening	Pressure pain at themandibular angle	Extraoral swelling	Mandibular border not palpable	Dysphagia	Dyspnea	Clunky language	Subfebrile temperature	Fever	Reduced general condition
Cheek	2	2 (100%)	0	1 (50%)	0	0	0	0	0	2 (100%)	2 (100%)
Floor of the mouth	5	1 (20%)	0	3 (60%)	1 (20%)	5 (100%)	0	3 (60%)	0	1 (20%)	3 (60%)
Fossa canina	2	0	0	2 (100%)	0	0	0	0	0	0	0
Masseterico-mandibular	2	2 (100%)	0	2 (100%)	0	1 (50%)	0	0	0	0	1 (50%)
Maxillary sinus	2	0	0	0	0	0	0	0	0	1 (50%)	1 (50%)
Maxillary sinus empyema	4	0	1 (25%)	1 (25%)	0	0	0	0	0	1 (25%)	1 (25%)
Paramandibular	1	0	0	1 (100%)	0	0	0	0	0	0	1 (100%)
Parotid gland	1	0	0	1 (100%)	0	0	0	0	0	0	0
Perimandibular	22	15 (68%)	3 (14%)	22 (100%)	22 (100%)	15 (68%)	1 (5%)	2 (9%)	4 (18%)	1 (5%)	15 (68%)
Peritonsillar and parapharyngeal	1	1 (100%)	0	1 (100%)	0	1 (100%)	0	0	0	0	1(100%)
Pterygomandibular	7	7 (100%)	2 (29%)	6 (86%)	0	5 (71%)	0	0	2 (29%)	1 (14%)	5 (71%)
Submandibular	10	6 (60%)	0	9 (90%)	1 (10%)	4 (40%)	0	1 (10%)	1 (10%)	1 (10%)	6 (60%)
Submental	3	0	0	3 (100%)	1 (33%)	2 (67%)	0	0	0	0	1 (33%)

While risk factors were not associated with age, older patients were more likely to have one (p = 0.008) or more than one (p = 0.006) comorbidity. The number of comorbidities was significantly correlated with the number of risk factors (p = 0.018). Men were more likely to have more risk factors than women (p = 0.006).

The mean hospital stay was 6.2 days (range 2 to 19 days). One patient was previously hospitalized and therefore excluded from the hospitalization analysis. 4 patients refused hospitalization. In total, 55 of the 60 patients were analyzed regarding factors influencing the length of inpatient treatment (Table 1 and Fig. 3). Longer length of inpatient stay correlated significantly with the likelihood of ICU admission (p < 0.001). 8 of the 60 patients (13.3%) required ICU admission, and 80% of the patients hospitalized for more than 7 days required ICU admission. 8 patients (13%) could not be extubated immediately after surgery and 5 patients (8%) required tracheostomy.

On the one hand, the number of microbes found in S and NS.

did not differ significantly and is summarized in Table 3. This was also the case for bugs found in both S and NTS. On the other hand, an analysis of the microbial species found in swabs and native samples showed distinct differences. Fungal spp. were found significantly more often in the native samples (p = 0.021). Streptococcus species, the most prevalent germ (n = 97), was detected more frequently in native samples (55 cases, 56.7%) than in swabs (42 cases, 43.3%). Similarly, fungal species (n = 23) were isolated more than twice as often in native samples (n = 16, 69.6%) compared to swabs (n = 7, 30.4%). Staphylococcus species (n = 15) were twice as common in swabs. In addition, Actinomycetes (n = 6) showed a distinct distribution, with 5 organisms detected in native samples and only one in swabs. Veillonellae (n = 4) were isolated exclusively from native samples. For the majority of other species, the difference in bug counts between swabs and native samples was minimal. In 13 of 60 cases, swabs did not yield detectable microbial growth. This was the case for native samples in 10 of 60 patients.

**Table 3:** Distribution of species in swabs (S) and native samples (NS)

Species	Number of microbiota						
	Prior antibiotic treatment (n = 18)		No prior antibiotic treatment (n = 42)		Overall (n = 60)		Total
	S	NS	S	NS	S	NS	
abiotrophia species	0	0	0	1	0	1	1
acinetobacter species	0	0	0	2	0	2	2
actinomyces species	0	1	1	4	1	5	6
candida species	1	5	6	11	7	16	23
capnocytophaga species	0	0	1	3	1	3	4
citrobacter species	0	0	1	1	1	1	2
clostridium species	0	0	0	1	0	1	1
cutibacterium species	0	0	3	3	3	3	6
eikenella species	2	3	3	2	5	5	10
enterobacter species	0	0	0	1	0	1	1
enterococcus species	0	0	1	2	1	2	3
escherichia species	1	1	0	2	1	3	4
fusobacterium species	1	4	7	7	8	11	19
gemella species	1	1	0	0	1	1	2
granulicatella species	1	0	0	1	1	1	2
klebsiella species	0	0	3	2	3	2	5
lactobacillus species	0	0	1	1	1	1	2
leptonema species	0	0	0	1	0	1	1
neisseria species	0	1	1	1	1	2	3
paenibacillus species	0	0	1	0	1	0	1
parvimonas species	4	5	9	5	13	10	23
peptostreptococcus species	1	0	0	0	1	0	1
porphyromonas species	0	1	0	0	0	1	1
prevotella species	1	5	20	12	21	17	38
rothia species	0	1	0	0	0	1	1
slakia species	1	0	1	2	2	2	4
staphylococcus species	1	0	9	5	10	5	15
streptococcus species	12	15	30	40	42	55	97
veillonella species	0	1	0	3	0	4	4
Total	27	44	98	113	125	157	282
	9,57%	15,6%	34,75%	40,07%	44,3%	55,7%	100%
	25,18%		74,82%		100%		

In 8 specimens, no pathologic microbiological species could be cultured in either the native or swab specimen. In these cases, microbial resistance testing (MRT) was not possible. MRT was performed in the remaining 52 cases. In 33.3% of the patients (n = 20), no resistance to microbial agents could be detected. 30% (n = 18) showed resistance to one and 23.3% (n = 14) to more than one antimicrobial agent. Antibiotic resistance is reported in Table 4.

**Table 4:** Antibiotic resistance

Group	Antibiotic	Frequency of resistance per patient (absolut and relative %)	Frequency of resistent species (absolut and relative %)	Number of resistent species (pathogen)
Lincosamide	Clindamycin	12/60 (20%)	17/62 (27,4%)	Streptococcus anginosus (3), Prevotella buccae (2), Streptococcus constellatus (2), Streptococcus intermedius (2), Streptococcus mitis/oralis (2), Streptococcus parasanguinis (2), Atopobium parvulum (1), Granulicatella adiacens (1), Prevotella denticola (1), Streptococcus sanguinis (1)
Peniciliine	Penicillin G	14/60 (23,3%)	17/62 (27,4%)	Prevotella buccae (5), Prevotella melaninogenica (2), Staphylococcus aureus (2), Neisseria mucosa (1), Parvimonas micra (1), Prevotella baroniae (1), Prevotella denticola (1), Prevotella intermedia (1), Prevotella nigrescens (1), Prevotella oris (1), Streptococcus mitis/oralis (1)
	Ampicillin	8/60 (13,3%)	11/62 (17,7%)	Escherichia coli (3), Klebsiella oxytoca (2), Klebsiella pneumoniae (2), Staphylococcus aureus (2), Staphylococcus epidermidis (1), Streptococcus mitis/oralis (1)
	Piperacillin	8/60 (13,3%)	8/62 (12,9%)	Escherichia coli (2), Klebsiella pneumoniae (2), Staphylococcus aureus (2), Klebsiella oxytoca (1), Staphylococcus epidermidis (1)
	Ampicillin/ Sulbactam	5/60 (8,3%)	6/62 (9,7%)	Escherichia coli (3), Citrobacter freundii (1), Enterobacter cloacae Komplex (1), Staphylococcus epidermidis (1)
	Piperacillin/Tazobactam	3/60 (5,0%)	3/62 (4,8%)	Escherichia coli (2), Staphylococcus epidermidis (1)
	Oxacillin	1/60 (1,7%)	1/62 (1,6%)	Staphylococcus epidermidis (1)
Cephalosporine	Ceftriaxon	6/60 (10,0%)	7/62 (11,3%)	Streptococcus constellatus (2), Abiotrophia defectiva (1), Escherichia coli (1), Staphylococcus epidermidis (1), Streptococcus mitis/oralis (1), Streptococcus parasanguinis (1)
	Cefuroxim	5/60 (8,3%)	6/62 (9,7%)	Enterococcus raffinosus (2), Escherichia coli (2), Enterococcus faecalis (1), Staphylococcus epidermidis (1)
	Cefpodoxim	3/60 (5,0%)	4/62 (6,5%)	Escherichia coli (2), Citrobacter freundii (1), Enterobacter cloacae Komplex (1)
	Cefotaxim	2/60 (3,3%)	2/62 (3,2%)	Escherichia coli (1), Streptococcus mitis/oralis (1)
	Cefazolin	1/60 (1,7%)	1/62 (1,6%)	Staphylococcus epidermidis (1)
	Ceftazidim	1/60 (1,7%)	1/62 (1,6%)	Escherichia coli (1)
Carbapeneme	Imipenem	1/60 (1,7%)	1/62 (1,6%)	Staphylococcus epidermidis (1)
	Meropenem	1/60 (1,7%)	1/62 (1,6%)	Staphylococcus epidermidis (1)
Fluorchinolone (II/III/IV)	Levofloxacin	4/60 (6,7%)	5/62 (8,1%)	Enterococcus raffinosus (2), Enterococcus faecalis (1), Escherichia coli (1), Staphylococcus epidermidis (1)
	Ciprofloxacin	4/60 (6,7%)	4/62 (6,5%)	Escherichia coli (2), Staphylococcus capitis (1), Staphylococcus epidermidis (1)
	Moxifloxacin	2/60 (3,3%)	2/62 (3,2%)	Escherichia coli (2)
o.G	Cotrimoxazol	6/60 (10,0%)	7/62 (11,3%)	Escherichia coli (3), Eikenella corrodens (1), Enterococcus faecalis (1), Enterococcus raffinosus (1), Granulicatella adiacens (1)
Nitroimidazole	Metronidazol	6/60 (10,0%)	7/62 (11,35)	Cutibacteriu acnes (4), Atopobium parvulum (2), Cutibacterium avidum (1)
Epoxid- Antibiotika	Fosfomycin	5/60 (8,3%)	6/62 (9,7%)	Staphylococcus capitis (3), Staphylococcus hominis (2), Prevotella buccae (1),
Makrolide	Erythromycin	5/60 (8,3%)	5/62 (8,1%)	Staphylococcus epidermidis (3), Staphylococcus hominis (1), Streptococcus mitis/oralis (1)
Aminoglykoside	Gentamicin	3/60 (5,0%)	3/62 (4,8%)	Granulicatella adiacens (1), Gemella species (1), Streptococcus intermedius (1)
o.G	Fusidinsäure	2/60 (3,3%)	2/62 (3,2%)	Staphylococcus epidermidis (1), Staphylococcus hominis (1)
Tetracycline	Doxycyclin	1/60 (1,7%)	1/62 (1,6%)	Staphylococcus epidermidis (1)

In terms of microbial species, 132 of the 194 species tested (68%) showed no resistance, whereas 62 species (32%) showed resistance to at least one antimicrobial agent.

**Fig. 4:**
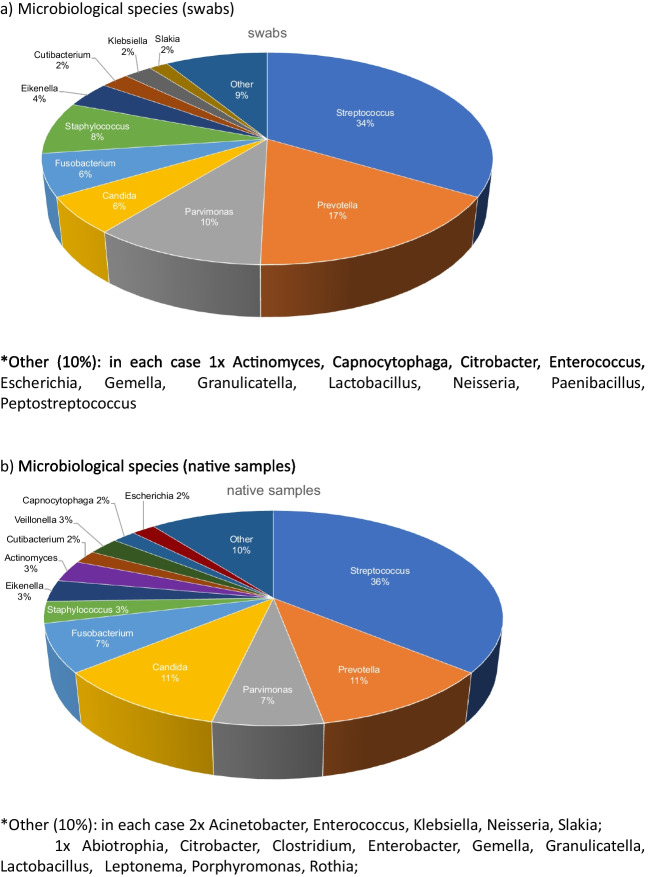

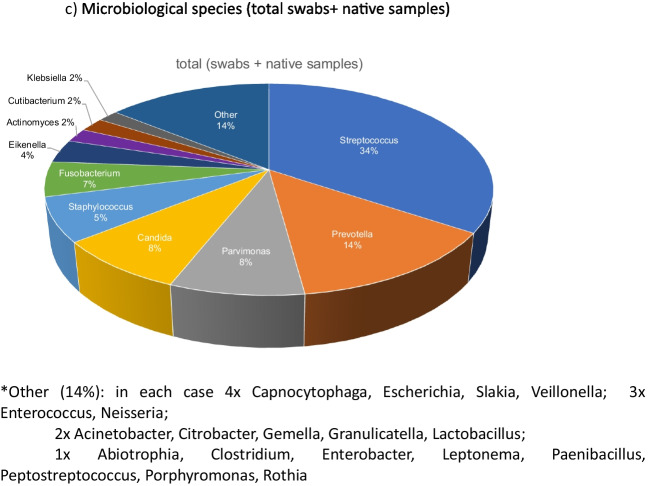
(**a**) Microbiological species (swabs) *Other (10%): in each case 1 × Actinomyces, Capnocytophaga, Citrobacter, Enterococcus, Escherichia, Gemella, Granulicatella, Lactobacillus, Neisseria, Paenibacillus, Peptostreptococcus (**b**) Microbiological species (native samples) *Other (10%): in each case 2 × Acinetobacter, Enterococcus, Klebsiella, Neisseria, Slakia; 1 × Abiotrophia, Citrobacter, Clostridium, Enterobacter, Gemella, Granulicatella, Lactobacillus, Leptonema, Porphyromonas, Rothia; (**c**) Microbiological species (total swabs + native samples) *Other (14%): in each case 4 × Capnocytophaga, Escherichia, Slakia, Veillonella; 3 × Enterococcus, Neisseria; 2 × Acinetobacter, Citrobacter, Gemella, Granulicatella, Lactobacillus; 1 × Abiotrophia, Clostridium, Enterobacter, Leptonema, Paenibacillus, Peptostreptococcus, Porphyromonas, Rothia

Among these microorganisms (n = 62), the highest prevalence of resistance was observed against clindamycin (27.4%) and penicillin (27.4%), followed by ampicillin (17.7%). Table 4 summarizes the percentage of antibiotic resistance in this study, while Fig. 4 illustrates the distribution of microbiota.

The presence of more than one resistance was significantly correlated with a longer duration of the hospital stay (p < 0.001), especially for stays longer than 7 days (p = 0.003). The presence of Gram-positive anaerobic bacteria was significantly correlated with a longer hospital stay (p = 0.027). Resistance to clindamycin correlated with longer hospital stay (p = 0.002) and longer ICU stays (p = 0.008). Among potential confounders, clindamycin resistance was significantly more common in women (p = 0.026). Metronidazole resistance was correlated with younger age (p = 0.024) and longer ICU stay (p = 0.001, n = 5). When analyzing the NTS, the presence of more than one risk factor was correlated with a higher number of microbes (p = 0.049) and Streptococci were found more frequently (p = 0.004). This was not the case for S (p = 0.212 and p = 0.439). Clindamycin resistance was detected in 12 patients, with a total of 17 resistant pathogens. Among ICU patients, 5 out of 8 (62.5%) had Clindamycin-resistant pathogens, and 3 of these had received Clindamycin preoperatively, in 2 cases due to penicillin allergy. Overall, 4 out of 12 patients (33.3%) with clindamycin resistance had received Clindamycin as initial therapy. Notably, 75% of these patients experienced prolonged hospital stays with ICU admission.

Metronidazole resistance was identified in 5 patients (7 resistant pathogens), including 2 ICU patients, but none of these patients had received metronidazole preoperatively.The presence of Gram-negative anaerobic bacilli in S correlated with increasing age (p = 0.028). This was not the case for NS and other bacterial groups.

Analysis of anesthesiological parameters showed that ASA 3 was associated with longer hospital stay, but the results were not statistically significant (p = 0.194). The Cormack-Lehane score, on the other hand, showed that higher scores (2 and 3) correlated with a higher likelihood of ICU stay (p = 0.007).

## Discussion

As predicted by the discoverer of penicillin, Sir Alexander Fleming, antimicrobial drug resistance has become a growing clinical problem, with an expected number of 10 million deaths per year by 2050 [25, 26].

**Fig. 5:**
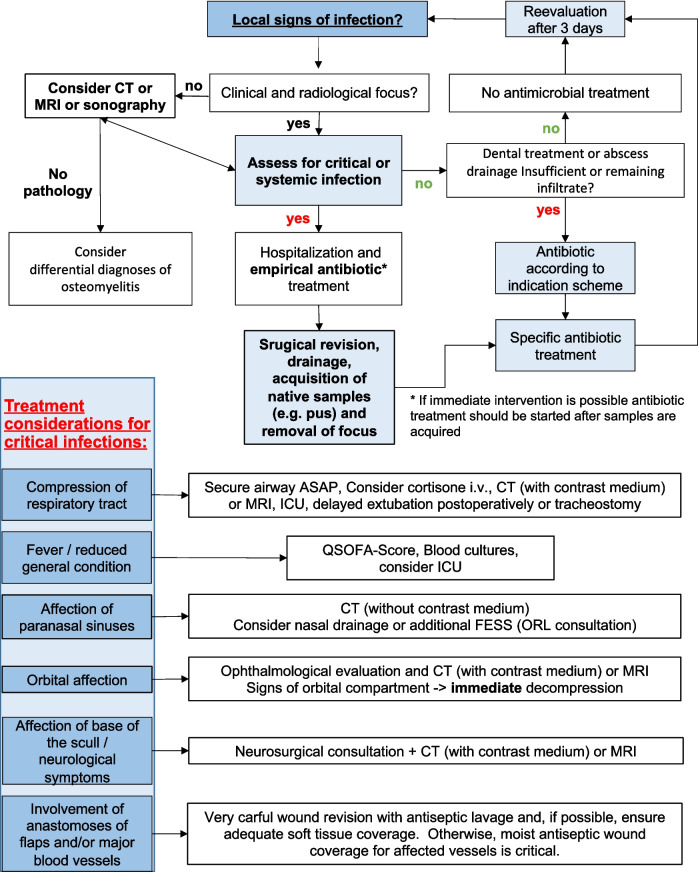
Management algorithm of acute oral and maxillofacial infections

In this study, we analyzed severe head and neck infections requiring hospitalization. Special emphasis was placed on the comparison of the microbiological analysis of NS and S in this context. However, the observations made in this study indicate potential areas for further refinement of current treatment strategies concerning critical and very critical infections. In particular, the role of native tissue sampling is not explicitly addressed in existing guidelines, despite its potential to provide a broader spectrum of microbial detection. Based on the successful management of the severe infections presented in this study, diagnostic and treatment options were suggested, as illustrated in Figs. 1 and 5.

Odontogenic infections are the most common cause of head and neck infections [27]. This is represented by the overwhelming majority of odontogenic foci in the study population. With very few exceptions, almost all cases in this study were in advanced but still moderate risk situations according to Jevon et. al [28]. Nevertheless, a significant number of patients required ICU admission. In particular, patients with delayed clinical improvement or persistent CRP and WBC elevations were more likely to require ICU care and advanced airway management. These findings underscore the importance of early diagnosis and treatment, with WBC serving as a key prognostic tool in non-responders.

More than two-thirds of these patients also presented initially in reduced general health.

However, consistent with the literature, no abscess specific abscess localization could be identified that was associated with a higher risk of complications or prolonged hospital stay [29].

The length of the hospital stay can be considered as a measure of the complexity and severity of the clinical situation and is associated with higher costs [30].

By streamlining diagnosis and treatment, complications could be reduced or even avoided, resulting in significant improvements in patient comfort and reduced burden to the health care system. Significant factors that increased the length of inpatient treatment were the need for ICU admission, the presence of Gram-positive anaerobic bacteria, and antibiotic resistance to more than one antimicrobial agent or to clindamycin, which also correlated significantly with the need for ICU admission. Resistance to metronidazole, on the other hand, correlated with a prolonged ICU stay. Like Heim et al., Wagendorf et al. recommended to reconsider the use of clindamycin as a first-line alternative to amoxicillin together with clavulanic acid after examining the microbiota in the sonicates of extracted teeth [14, 15]. The percentage of antibiotic resistance to clindamycin in the present study was as high as that of conventional penicillin and comparable to the literature [6, 14, 15, 31]. This could be explained by its excessive use in dental and maxillofacial clinics over the years [32]. The presented findings therefore suggest a potential association between Clindamycin resistance and disease severity, particularly in ICU patients. Given the significant impact of Clindamycin resistance, careful evaluation of reported penicillin allergies is crucial, as misdiagnosis may lead to suboptimal antibiotic therapy and worse outcomes. The prevalence of metronidazole resistance is reported to be comparatively low and its impact is not yet fully understood [33]. However, the emergence of multidrug resistant anaerobes is an increasing clinical challenge and studies using 16S rRNA PCR revealed an underrepresentation of anaerobic bacteria in cultures [33–35]. This is underlined by the fact that resistance to more than one antibiotic correlates significantly with longer hospital stays. In the present study, the presence of Gram-positive anaerobes in particular led to significantly longer hospital stays. The vast majority of these were Parvimonas micra spp., which are particularly associated with biofilm formation and are part of the so-called periodontopathogenic “orange complex” and could be considered as precursors for severe disease [36, 37].

In addition, compromised host defenses and immunocompetence may reduce physiologic reserve and prolong recovery and hospitalization [28, 38]. In the study cohort, risk factors were significantly correlated with medical conditions, which in turn increased with age. In a selected patient population with severe head and neck infections, it is noteworthy that almost half of the patients were smokers and 10% of the patients admitted using other drugs. Although the proportions of smokers in the group with a significantly prolonged hospitalization (more than 7 days) was 60%, these differences were not statistically significant. However, the proportion of smokers in the German population is about 20%, which is significantly lower than in the study population [39]. Therefore, smoking status, substance abuse, age, and multiple comorbidities can be considered risk factors for a more complicated course and longer hospital stay [28, 38, 40].

Although the microbiologic literature is clear that native specimens are preferable to swab specimens, the clinical reality and many clinical studies focus on swab specimens as the primary sampling tool for head and neck abscesses [14, 16, 19, 22, 23, 41–43]. Zhen et al. and Trimarchi et al. included native samples (aspirates/pus specimens) but did not differentiate between S and NS [5, 44].

Overall, the microbial spectrum in S and NS was similar, with no significant differences between the two. However, some differences may be clinically relevant. Considering that S and NS were collected from the same area, it is noteworthy, that five NS showed the growth of Actinomyces, while only one corresponding S could provide the same finding. Similarly, Veillonella was found in 4 NS but none of the S. In contrast, Staphylococci were found in twice as many S as NS, suggesting a sample dependent pathogen selection or potential contamination.

The American guidelines for the use of microbiological laboratory tests specifically state that it is always better to provide as much of the actual specimen in different portions as possible rather than swabbing the specimen [17]. This can be especially helpful in the case of suspected atypical organisms and fungi [17, 45]. This was reproducible in this study, as NS were significantly more likely to provide evidence of Candida spp., which could be of importance in immunocompromised or irradiated [46]. Since obtaining reliable native tissue samples from the depth of a cervical abscess poses a risk of injuring adjacent structures, a"brush biopsy"of the abscess membrane could be a valid alternative for tissue sampling [47].

Especially in life-threatening situations or organs at risk (e.g., eyes), early and decisive treatment is critical, including consideration of the potential contribution of atypical microbiological organisms [17, 31]. Actinomyces infections are common in patients with frequent antibiotic use due to the slow growth of the bacteria and natural resistance to short-term therapy, as often seen in drug-related osteonecrosis of the jaw [2, 48, 49].

Veillonella spp. facultative anaerobic cocci are important components of the root canal infection microbiota and commensals, but their role as a pathogens remains elusive [50–54]. New methods like microbiological analysis using next generation sequencing might further enhance the detection of these pathogens to clarify their significance [55]. In this study, however, the presence of Veilonella was not correlated with adverse outcome. Although these were advanced cases with potentially life-threatening conditions, all patients survived without serious or permanent organ damage. Nevertheless, some complications, such as airway obstruction requiring ICU monitoring or tracheostomy, could have led to this outcome, highlighting the need for a systematic approach to the management of these infections. Therefore, intended derived a comprehensive algorithm to streamline the diagnosis and treatment (Fig. 5).

Since a delayed or incorrect initial diagnosis can have devastating consequences, we intended to provide the following systematic approach.

By classifying cranio-maxillofacial infections into green (non-critical), yellow (critical), and red (very critical) according to a risk-level indicator system, we aim to facilitate decision-making by applying the traffic-light color code.

Common findings for non-critical infections indicate a local limitation with little or no systemic response and no involvement of critical regions, as shown in Fig. 1. Antibiotic treatment may be indicated if a tendency to spread is observed [56, 57]. Critical infections, on the other hand, are associated with systemic involvement, such as decreased general condition and/or fever, and are characterized by their potential for rapid progression and severe complications, including airway obstruction, sepsis, and mediastinitis [6, 57, 58]. Involvement of critical regions, such as the orbit or angular vein involvement may also qualify the patient for hospitalization [6, 59, 60].

In these cases, intravenous antibiotic treatment is indicated [6, 60]. However, surgical drainage and microbiologic specimens should be obtained as soon as possible before the use of antimicrobial agents. Highly critical infections are those that threaten life or critical organs or functions such as the eyes [60]. Skull base involvement and intracranial spread are other examples of very critical infections that do not allow for undue delay [17, 61]. These situations, such as impeding septic shock, may require immediate and broad-spectrum antimicrobial treatment, especially if immediate surgical intervention is not possible, and fungi and atypical microorganisms, like mycobacteria, need to be considered and treated as appropriate [62]. Very critical infections also generally require a multidisciplinary approach, involving departments according to the area affected, as well as ICU consultation [6, 7, 44, 57]. According to the clinicians, microvascular flaps can also be considered as vital or critical organs, since the loss of a flap can severely alter the function and quality of life of the affected patient and ultimately lead to the need for another major operation [63].

In addition to local factors, the initial physical examination must address general medical, neurological, and ophthalmologic functions. Patient-specific characteristics must be taken into account, as these may significantly alter the risk of an adverse outcome. Particular attention must be paid to the possibility of developing or incipient airway obstruction, and early anesthesia consultation is important, as the results of this study showed that higher ASA and Cormack-Lehane-Scores were associated with longer hospital stay and higher likelihood of ICU monitoring, respectively.

Potential drawbacks of this study include its pilot nature and the therefore limited number of cases, as well as the heterogeneity of the head and neck regions. The study relies on culture-dependent methods, as molecular biological techniques are not yet fully established in routine clinical practice. The primary aim was to assess the clinical relevance of different sampling techniques under standard conditions. In addition, native samples were not transported in specific anaerobic media, which may have given and advantage to swabs. Nevertheless, native samples did not show inferior results, revealing significantly more Candida spp. and higher yields of Actinomyces and Veilonella spp.

## Conclusion

Severe head and neck infections require a comprehensive diagnostic and treatment protocol to manage these potentially life-threatening conditions. The frequent use of clindamycin as a first-line alternative to amoxicillin with clavulanic acid needs to be seriously reconsidered. Surgical incision and drainage remain the cornerstone of treating odontogenic abscesses. However, in critical infections, targeted antimicrobial therapy is an essential adjunct. While routine collection of native tissue samples (NS) is not universally supported by our data, the observed differences in microbial composition suggest potential benefits in selected cases. The increasing resistance to clindamycin highlights the need for a risk-adapted antibiotic strategy rather than empirical prescribing. Brush biopsies for microbiological sampling might be a valid alternative, combining the advantages of both methods, and warrant further investigation.

## Data Availability

The data that support the findings of this study are not openly available due to reasons of sensitivity and are available from the corresponding author upon reasonable request.

## Declarations

### Clinical trial number

This study was registered in the German Clinical Trial Register under the clinical trial number DRKS00036162.
